# Editorial: Plant derived bioactive compounds in the management and treatment of metabolic syndrome

**DOI:** 10.3389/fphar.2024.1423125

**Published:** 2024-06-18

**Authors:** Kamran Ashraf, Abul Kalam Najmi, Nafees Ahemad

**Affiliations:** ^1^ Faculty of Pharmacy, Cawangan Selangor, Kampus Puncak Alam, Universiti Teknologi MARA(UiTM), Puncak Alam, Selangor Darul Ehsan, Malaysia; ^2^ Department of Pharmacology, School of Pharmaceutical Education and Research, Jamia Hamdard (Hamdard University), New Delhi, India; ^3^ School of Pharmacy, Monash University Malaysia, Petaling Jaya, Selangor, Malaysia

**Keywords:** metabolic syndrome, bioactive compounds, diabetes mallitus, cardiovascular disease, obesity

Metabolic syndrome refers to a group of interrelated conditions that have been associated with the onset of cardiovascular diseases, type 2 diabetes, cognitive decline, and abnormal renal function. There is a substantial risk of cardiovascular disease and early mortality with these co-occurring diseases. The first line of treatment for metabolic syndrome is changing one’s lifestyle, which includes modifying food choices and increasing physical activity. Early treatment side effects associated with synthetic medication therapy for metabolic syndrome include altered renal enzyme functioning, gastrointestinal distress, flatulence, and hepatic abnormalities. A correct lifestyle and a balanced diet, not unbalanced towards an excess of simple sugars or saturated fats, can help prevent the dysmetabolisms underlying the metabolic syndrome. Likewise, a great contribution can come from appropriate and effective integration with bioactive substances present in certain foods or in food supplement formulations.

The present Research Topic of papers is based on the therapeutic significance and translational value of some of the bioactive in metabolic and related disorders.

Wang et al. have investigated the effect of proanthocyanidines (BLPs) at different doses on glucose uptake and glucose transport in human intestinal epithelial cells (Caco2 cells), The results showed that BLPs significantly decreased glucose uptake and disaccharidase activity. It was attributed to the suppression of glucose transporter 2 (GLUT2) and sodium-dependent glucose cotransporter 1 (SGLT1) by BLPs. BLPs were found to significantly downregulate the transcription level and protein expression of glucose transporters (*p* < 0.05). These results suggest that BLPs inhibit intestinal glucose transport via inhibiting the expression of glucose transporters. It indicated that BLPs could be potentially used as a functional food in the diet to modulate postprandial hyperglycemia. The review by Yang et al. have collected information about the classical Yao-Shan of TCM (Traditional Chinese Medicine) in the treatment and management of metabolic diseases. They enhanced the current progress of some Yao-Shan of TCM with modern medicine strategies. They also tried to uncover the mystery of Yao-Shan of TCM through modern biological and chemical strategies that might help and open a door to modulating metabolic homeostasis and diseases. Xu et al. have explored the pharmacological mechanism of Dai-Zong-Fang (DZF) against obesity. *In vivo* diet-induced obesity (DIO) model was established by feeding high-fat diets to C57BL/6J mice. The findings imply that DZF can increase browning of WAT, attenuate obesity, and lessen abnormalities associated with glucose and lipid metabolism by activating the PKA pathway. This suggests that DZF may be chosen as an anti-obesity medication to help obese patients. Sun et al. in their review article, have explored TCM and related active compounds in the treatment of gout. Numerous TCM-based therapies and associated active ingredients have proven effective in controlling gout, expertly controlling serum uric acid (UA) levels, and slowing the progression of inflammation. This review gathers important basic information on the UA transporters and molecular signalling pathways associated with gout that are regulated by TCM. Shi et al. have highlighted the importance of the effectiveness of Shuxuening injections in the treatment of coronary heart disease. The study showed that combination therapy increased clinical efficacy and decreased the frequency and duration of angina.

The review by Deng et al. highlights the importance of Rhein in the treatment of diabetes mellitus. Rhein, the main active component of Rhubarb, is a 1, 8-dihydroxy anthraquinone derivative. This study shows that rhein can prevent and treat diabetes by ameliorating IR, anti-inflammatory and anti-oxidative stress, and protecting islet cells, which provides a theoretical basis for further application of rhein.

In conclusion, bioactive substances present in certain foods or in food supplement formulations can help patients lose weight, improve their cardiovascular health, lower their blood pressure, and enhance their glucose metabolism, all of which are good for improving health and thus help in the treatment and management of metabolic syndrome.

